# Whole genome sequence-based characterisation of Shiga toxin-producing *Escherichia coli* isolated from game meat originating from several European countries

**DOI:** 10.1038/s41598-023-30333-4

**Published:** 2023-02-24

**Authors:** Magdalena Nüesch-Inderbinen, Andrea Treier, Marc J. A. Stevens, Roger Stephan

**Affiliations:** Institute for Food Safety and Hygiene, Vetsuisse Faculty, University of Zurich, Zurich, Switzerland

**Keywords:** Microbiology, Bacterial genes

## Abstract

Game meat is becoming increasingly popular but may be contaminated with pathogenic bacteria such as Shiga toxin-producing *Escherichia coli* (STEC). STEC cause gastrointestinal illnesses including diarrhoea, haemorrhagic colitis (HC), and the haemolytic uremic syndrome (HUS). The aim of this study was to assess the occurrence of STEC in 92 meat samples from chamois (n = 2), red deer (n = 27), roe deer (n = 38), and wild boar (n = 25), from Switzerland and other European countries. After enrichment, Shiga-toxin encoding genes (*stx*) were detected by PCR in 78 (84%) of the samples and STEC were isolated from 23 (25%) of the same samples. Nine different serotypes and eight different sequence types (STs) were found, with O146:H28 ST738 (n = 10) and O110:H31 ST812 (n = 5) predominating. None of the STEC belonged to the so-called top-five serogroups O26, O103, O111, O145, and O157. Subtyping of *stx* identified *stx1c* (n = 9), *stx2a* (n = 1), *stx2b* (n = 19), *stx2e* (n = 2), and *stx2g* (n = 1). Additional virulence factors (VFs) comprised *ehx* (n = 12), *iha* (n = 21), *sta1* (n = 1), and *subAB* (n = 19). None of the isolates contained the *eae* gene. Twenty-one STEC contained VFs associated with extra-intestinal pathogenic *E. coli* (ExPEC). Overall, the pathogenic potential of STEC in game meat is moderate, though the isolation of one STEC strain carrying *stx2a*, and of STEC/ExPEC hybrids suggests a role of game meat as a potential source of STEC infections in humans. Therefore, detailed knowledge of the safe handling and preparation of game meat is needed to prevent foodborne infections.

## Introduction

Shiga toxin-producing *Escherichia coli* (STEC) cause an estimated 2.8 million acute illnesses annually, representing one of the most common causes of gastrointestinal illness worldwide^1^. STEC may cause mild to severe non-bloody or bloody diarrhea (BD), haemorrhagic colitis (HC), and the life-threatening haemolytic uremic syndrome (HUS)^2^. STEC are characterized by two types of Shiga toxins encoded by *stx1* and *stx2*, with four *stx1* (*stx1a*, *stx1c*, *stx1d, and stx1e*) and 14 *stx2* (*stx2a*-*stx2m, and stx2o*) subtypes described so far^3–6^. STEC harbouring *stx2a* and *stx2d* are understood to be associated with severe disease whereas STEC carrying *stx2b* and *stx2e* are typically linked to mild clinical symptoms or asymptomatic faecal carriage^7,8^. Other *stx2* subtypes including *stx2f*, *stx2g*, *stx2m* and *stx2o* are infrequently identified in STEC from human samples, but *stx2f*-positive strains have been isolated from patients with HUS^5^. Furthermore, many STEC strains feature additional virulence genes encoding toxins and adherence factors such as *astA* (enteroaggregative *E. coli* heat-stable toxin 1), *eae* (adherence factor intimin), *ehxA* (enterohemolysin), *iha* (IrgA homolog adhesin), *lpf* (long polar fimbriae), and *subAB* (subtilase cytotoxin)^9,10^. Moreover, STEC may also exhibit virulence properties from other *E. coli* pathotypes such as enteroaggregative *E. coli* (EAEC) or extraintestinal pathogenic *E. coli* (ExPEC), for example the STEC/EAEC hybrid serotype O104:H4 that caused the major HUS outbreak in Germany in 2011^11,12^, or the STEC/ExPEC hybrid serotype O80:H2 which has emerged in France and Switzerland as a serogroup causing HUS and bacteraemia^13–15^.

Although frequently linked to food borne outbreaks, the majority of STEC infections remain sporadic and are significantly associated with person-to-person transmission, contact with animals or their environment, and consuming undercooked or raw meat, in particular beef^16–18^.

Meat from wild game is gaining in popularity in many countries as it appeals to a growing demand for foods that are nutritious and serve as an alternative to conventional meat from intensive livestock production^19^. Despite the growing interest in meat from game animals, European legislation (Commission Regulation (EC) No. 2073/2005) does not specify hygiene criteria for raw wild game meat regarding STEC, and information on the prevalence and pathogenicity of STEC in this food category is limited^20^.

The aim of this study was therefore to assess the occurrence of STEC in meat samples of chamois (n = 2), red deer (n = 27), roe deer (n = 38), and wild boar (n = 25) originating from Switzerland and other European countries, and to analyse the STEC isolates for serotypes, multilocus sequence types, and virulence gene content, using a whole genome sequencing approach.

## Results

### Real-time screening for *stx* genes and isolation of STEC

Using real-time PCR screening identified *stx1* and/or *stx2* in 77 (84%) of the 92 game meat samples analysed in this study. Thereof, the majority (75 of 77 samples) contained *stx2*, alone or in combination with *stx1*. The positive samples included one of the two chamois, 24 of the 27 red deer, 36 of 38 roe deer, and 16 of 25 wild boar meat samples (Table 1).

**Table 1:** Detection of *stx* genes by PCR and isolation of Shiga toxin-producing *Escherichia coli* (STEC) strains from 92 samples of game meat.

Type of meat	No. samples	Molecular detection of *stx* genes				Isolation of STEC strains^a^	
		No. *stx*-positive samples	No. of samples (%) positive for			No. STEC-positive samples	No. STEC isolated
			*stx1*	*stx2*	*stx1* and *stx2*		
Chamois	2	1	1	0	0	1	1
Red deer	27	24	0	16	8	4	4
Roe deer	38	36	0	9	27	15	19
Wild boar	25	16	1	12	3	3	3
Total	92	77	2	37	38	23	27

^a^PCR positive samples were further cultured and at least one STEC was isolated by growth on Brolacin agar or CHROMagar™. For details see text.

STEC were isolated from 23 of the 77 *stx*-positive real-time PCR samples, corresponding to a recovery rate of 30% and an overall STEC prevalence of 25%. The roe deer meat samples W42, W96, W98, and W99 contained two distinct STEC isolates resulting in a total of 27 STEC available for further analysis (Table 1).

### Serotypes, multilocus sequence types (MLST) and phylogenetic relationship

**Figure 1:**
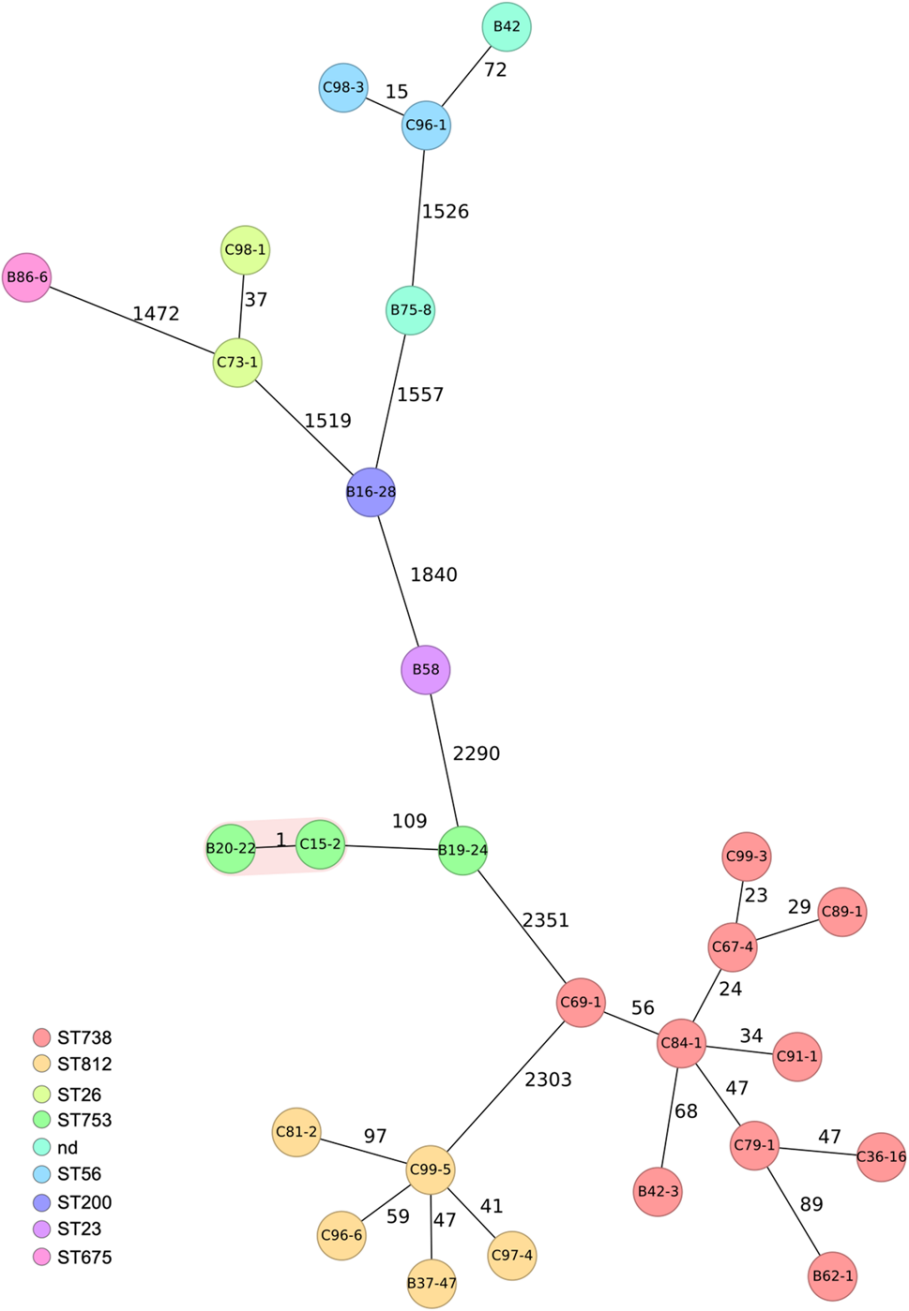
Phylogenetic relationship of 27 Shiga toxin-producing *Escherichia coli* (STEC) isolated from game meat based on their core genome multilocus sequence type (cgMLST) allelic profiles. The minimum spanning tree was generated using SeqSphere (Ridom GmbH). Numbers on connecting lines indicate the number of allele differences between two strains. The colors of the circles represent STs according to the Warwick scheme (http://enterobase.warwick.ac.uk). Strain IDs are indicated in the circles.

Overall, nine different serotypes were identified among the 27 STEC (Table 2). O146:H28 and O110:H31 were the predominant serotypes, accounting for 10 (37%), and 5 (19%) of all STEC isolates (Table 2). Eight sequence types (STs) were assigned among the 27 STEC, thereof, ST738 (n = 10) and ST812 (n = 5) were predominant. Isolates with the same serotype were assigned to the same sequence type, with the exceptions of one STEC O21:H21 (isolate B42 recovered from roe deer meat), and STEC O179:H8 (isolate B75-8 recovered from wild boar meat), which were both not assigned to any ST (Table 2). The population structure of the strains was visualized by means of a cgMLST-based phylogenetic tree. The isolates grouped according to serotypes and STs (Fig. 1). Except for two STEC O27:H30 (B20-22 recovered from red deer from Slovenia, and C15-2 from red deer from Switzerland, respectively), they were phylogenetically clearly distinct, with ≥ 15 different alleles between each pair of neighboring isolates (Fig. 1).

**Table 2:** Characteristics of 27 Shiga toxin-producing *Escherichia coli* (STEC) isolated from game meat from different animals and countries.

Strain ID	Sample ID	Animal species	Country of origin	Supplier	Serotype	ST	*stx 1*	*stx2*	Other virulence factor genes
B58	W58	Red deer	Hungary	Plant	O8:H9	23	−	*stx2e*	*hra, iss, lpfA, ompT, terC, traT*
B42	W42	Roe deer	Poland	Plant	O21:H21	nd	−	*stx2b*	*astA, espI, gad, iha, ireA, iss, lpfA, ompT, subAB2, terC, traT*
C96-1	W96	Roe deer	Switzerland	Hunter	O21:H21	56	−	*stx2b*	*astA, fyuA, gad, iha, ireA, iss, kpsE, lpfA, ompT, subAB2, terC, traT*
C98-3	W98	Roe deer	Switzerland	Hunter	O21:H21	56	−	*stx2b*	*astA, fyuA, gad, iha, ireA, iss, kpsE, lpfA, ompT, subAB2, terC, traT*
B19-24	W19	Red deer	Slowenia	Plant	O27:H30	753	−	*stx2b*	*astA, eilA, gad, hra, iha, ireA, iss, ompT, subAB2, terC, traT*
C15-2	W15	Red deer	Slowenia	Plant	O27:H30	753	−	*stx2b*	*air, chuA, eilA, gad, iha, ireA, iss, ompT, subAB2, terC, traT*
B20-22	W20	Red deer	Switzerland	Butcher	O27:H30	753	−	*stx2b*	*air, chuA, eilA, gad, iha, ireA, iss, ompT, subAB2, terC, traT*
B86-6	W86	Chamois	Switzerland	Hunter	O76:H19	675	*stx1c*	*stx2b*	*ehxA, gad, iha, ireA, kpsE, kpsE, lpfA, pic, senB, sitA, subAB2, terC, terC, traT*
C81-2	W81	Roe deer	Switzerland	Butcher	O110:H31	812	*stx1c*	−	*chuA, focC, fyuA, gad, hra, ireA, iroN, irp2, ompT, papC, pic, sfaD, terC, traT, vat, yfcV*
B37-47	W37	Roe deer	Switzerland	Hunter	O110:H31	812	*stx1c*	*stx2b*	*celb, chuA, fyuA, gad, hra, ireA, irp2, ompT, papC, pic, terC, traT, yfcV*
C96-6	W96	Roe deer	Switzerland	Hunter	O110:H31	812	*stx1c*	−	*astA, chuA, fyuA, gad, hra, irp2, ompT, papC, terC, traT, yfcV*
C97-4	W97	Roe deer	Switzerland	Hunter	O110:H31	812	*stx1c*	−	*chuA, fyuA, gad, hra, ireA, irp2, kpsE, ompT, papC, pic, subAB2, terC, yfcV*
C99-5	W99	Roe deer	Switzerland	Hunter	O110:H31	812	*stx1c*	*stx2b*	*chuA, fyuA, gad, hra, iha, ireA, irp2, kpsE, ompT, papC, pic, subAB2, terC, traT, yfcV*
B62-1	W62	Roe deer	Germany	Retailer	O146:H28	738	−	*stx2b*	*astA, chuA, ehxA, hra, iha, ireA, iss, lpfA, ompT, senB, subAB2, terC, traT, usp*
B42-3	W42	Roe deer	Poland	Plant	O146:H28	738	−	*stx2b*	*astA, chuA, hra, iha, ireA, iss, lpfA, ompT, subAB2, terC, traT, usp*
C84-1	W84	Roe deer	Switzerland	Butcher	O146:H28	738	−	*stx2b*	*astA, chuA, ehxA, hra, iha, ireA, iss, lpfA, ompT, senB, subAB2, terC, traT, usp*
C69-1	W69	Roe deer	Switzerland	Butcher	O146:H28	738	−	*stx2b*	*astA, chuA, ehxA, gad, hra, iha, iss, lpfA, ompT, terC, traT, usp*
C36-16	W36	Roe deer	Switzerland	Hunter	O146:H28	738	*stx1c*	*stx2b*	*astA, chuA, hra, iha, ireA, iss, lpfA, ompT, terC, traT, usp*
C67-4	W67	Roe deer	Switzerland	Hunter	O146:H28	738	−	*stx2b*	*astA, chuA, ehxA, hra, iha, ireA, iss, lpfA, ompT, subAB2, terC, traT, usp*
C91-1	W91	Wild boar	Switzerland	Hunter	O146:H28	738	−	*stx2b*	*astA, chuA, ehxA, hra, iha, ireA, iss, lpfA, ompT, senB, subAB2, terC, traT, usp*
C99-3	W99	Roe deer	Switzerland	Hunter	O146:H28	738	−	*stx2b*	*astA, chuA, ehxA, hra, iha, ireA, iss, lpfA, ompT, senB, subAB2, terC, traT, usp*
C79-1	W79	Roe deer	Switzerland	Butcher	O146:H28	738	−	*stx2b*	*astA, chuA, gad, hra, iha, ireA, iss, lpfA, ompT, subAB2, terC, traT, usp*
C89-1	W89	Wild boar	Switzerland	Hunter	O146:H28	738	−	*stx2b*	*astA, chuA, ehxA, hra, iha, ireA, iss, lpfA, ompT, senB, subAB2, terC, traT, usp*
B75-8	W75	Wild boar	Switzerland	Butcher	O179:H8	nd	−	*stx2a*	*celb, ehxA, epeA, espP, gad, hra, iha, iss, lpfA, ompT, subAB1, terC, traT*
B16-28	W16	Red deer	Austria	Plant	O187:H28	200	−	*stx2g*	*astA, ehxA, gad, lpfA, sta1, terC, traT*
C73-1	W73	Roe deer	Switzerland	Butcher	Ond:H8	26	*stx1c*	−	*astA, celb, ehxA, gad, iha, lpfA, subAB2, terC, traT*
C98-1	W98	Roe deer	Switzerland	Hunter	Ond:H8	26	*stx1c*	−	*astA, ehxA, iha, iss, lpfA, ompT, senB, terC, traT*

nd, not determined; ST, sequence type; +, presence of gene(s); −, absence of gene(s).

### Stx subtypes and additional virulence determinants

Subtyping of the *stx* genes revealed that five of the 27 isolates (19%) harboured *stx1c* only (Table 2). Eighteen of 27 (67%) carried *stx2* genes only; i. e. *stx2a* (n = 1), *stx2b* (n = 15), *stx2e* (n = 1), and *stx2g* (n = 1). Four of 27 (15%) harboured the combination of *stx1c* and *stx2b* genes (Table 2). The *stx2a* gene, which is associated with severe disease was identified in the O179:H8 isolate B75-8 recovered from wild boar meat.

Besides *stx* genes, a large number of additional virulence genes were identified among the strains, including genes encoding toxins *astA* (n = 18)*, ehxA* (n = 12)*, eilA* (n = 3)*, subAB1* (n = 1)*, subAB2* (n = 18)*, sta1* (n = 1)*, usp* (n = 10)*,* and *vat* (n = 1), and adhesins (*air* (n = 2)*, espI* (n = 1)*, espP* (n = 1)*, focC* (n = 1)*, hra* (n = 18)*, iha* (n = 21)*, lpfA* (n = 19)*, ompT* (n = 24)*, papC* (n = 5)*, pic* (n = 5)*, sfaD* (n = 1)*,* and *yfcV* (n = 5). The adhesin gene *saa* was found only as a partial sequence in one isolate (B75-8) (data not shown).

Further VFs included those associated with iron acquisition *fyuA* (n = 7)*, ireA* (n = 20)*, iroN* (n = 1)*, irp2* (n = 5)*, and sitA* (n = 1), with the ability to survive bactericidal serum activity (*iss,* n = 19), those associated with acidic tolerance (*gad,* (n = 17), a gene encoding the capsule polysaccharide export protein (*kpsE,* n = 5), an outer membrane protein complement resistance gene (*traT,* n = 26), and the tellurite resistance gene *terC* (n = 27). Notably, none of the isolates in this study carried the *eae* gene, which is an adhesin that is present in many STEC^21^.

Some of the VFs mentioned above, including *air, eilA,* and *pic*, are also associated with EAEC^22^. However, the *aggR* and the *aat* genes, which are typical molecular predictors of EAEC^9^, were not detected.

The presence of *sta1*, encoding the heat stable enterotoxin typically produced by ETEC^23^, was detected in the O187:H28 isolate B16-28 from red deer meat (Table 2).

Additionally, many of the VFs found among the isolates are associated with ExPEC including c*huA* (n = 18), *focC* (n = 1), *fyuA* (n = 7), *ireA* (n = 20), *irp2* (n = 5), *papC* (n = 5), *sfaD* (n = 1), *usp* (n = 10), *vat* (n = 1), and *yfcV* (n = 5) (Table 2).

### Antimicrobial resistance genes

All 27 STEC isolates in this study harboured *bla*_EC_ genes, which are Ambler class C cephalosporinases derived by mutations from *ampC*^24^ (data not shown). Further, all isolates contained genes for the *E. coli* resistance-nodulation-division (RND) efflux pump AcrAB-TolC which is a major contributor to intrinsic resistance to antibiotics and resistance to bile salts which allows bacterial colonization and adaptation to the intestinal tract^25^ (data not shown).

## Discussion

While recent years have seen an increase in the popularity of game meat, there is a concern that this comes with the risk of exposure to zoonotic pathogens, including STEC. STEC constitute part of the microbiota of the gastrointestinal tract of a variety of wild animals and may contaminate the meat during evisceration and skinning procedures, processing, and packaging^26,27^.

In this study, the presence of *stx1* and *stx2* genes was detected in 84% of the enrichment cultures, indicating that the overall contamination of game meat with STEC is high. In 23 of the *stx*-positive meat samples STEC could be isolated. Therefore, with an overall prevalence of 25%, the level of STEC contamination in the present study was considerably higher than the 5.6% STEC prevalence reported in game meat and game meat products in Spain during 2009–2010 and 2010–2011^28^, the 9.9% prevalence in retail game meat from Germany in 2006^29^, and the 10% prevalence in red deer meat samples from the USA in 2013^30^. However, comparative data are still scarce and differences in the testing methodologies of different studies may lead to variations between the results. Nevertheless, the present study provides evidence that the occurrence of STEC in game meat may currently be underestimated.

With one exception (isolate B75-8 O179:H8 harbouring *stx2a*), none of the isolates contained the virulence genes *stx2a*, *stx2d*, or *eae*, all of which are significantly associated with severe disease in humans^8,31^. Further, none of the isolates belonged to the so called “top five” serogroups of human pathogenic STEC O157, O26, O103, O111, and O145^5^, indicating that overall, the pathogenic potential of STEC in game meat is rather low. Nonetheless, other toxin genes, including *astA*, *ehxA*, *subAB1* and *subAB2 f*ound in 18/27, 12/27, 1/27, and 18/27 of the STEC in this study, are also considered important virulence markers for STEC pathogenesis and are frequently detected among human clinical isolates^32,33^. Notably, the subtilase cytotoxin subtype SubAB2 is an emerging pathogenic factor that is prevalent among human *eae*-negative STEC and also typically found among STEC from wildlife and small ruminants^33–35^. Moreover, the majority of the isolates (21 of the 27) harboured *iha*, which is thought to contribute to pathogenicity of *eae*-negative STEC by facilitating attachment to intestinal cells^36^. These findings indicate that the STEC occurring in game meat have the potential to cause disease in humans. Notably, STEC O187:H28 ST200 (isolate ID B16-28 recovered from red deer meat) carrying the rare *stx2g* subtype co-harboured the *sta1* gene, a heat stable enterotoxin typically produced by ETEC. Similar hybrid STEC/ETEC O187:H28 have been described recently from free-ranging red deer in Italy^37^, from flour samples in Germany and Switzerland^38,39^, and from a small child with diarrhoea in Sweden^40^. This highlights the importance of hybrid STEC and shows that game meat might serve as vehicle for possible human STEC/ETEC infections.

Interestingly, 24 out of 27 (89%) isolates harboured one or more virulence factors which are characteristic of ExPEC^41–43^. Although infections with the majority of the STEC in this study are less likely to cause severe gastrointestinal symptoms, STEC/ExPEC should be not underestimated due to the possibility of a systemic infection in combination with gastrointestinal disease^44^.

The most frequently identified STEC serotype in the present study was O146:H28 (ST738) harbouring *stx2b*. STEC O146 is among the most common non-O157 serogroups associated with human illness in Europe^5^, and STEC O146:H28 harbouring *stx2b* were found in 4% of all human non-O157 STEC infections in Switzerland in 2017^32^. STEC O146:H28 has also been identified in raw dog food^45^ and hulled wheat and rye flour samples in Switzerland^46^, indicating its wide distribution throughout various ecological niches. As was seen for the majority of the STEC in this study, STEC O146:H28 carried a range of VF associated with extraintestinal pathogenic disease, but was the only serotype to harbour the uropathogenic-specific protein (*usp*) gene which has been described in *E. coli* that are linked to pyelonephritis, prostatitis and bacteraemia^47^. Other STEC described in this study are not commonly associated with human disease but have been recovered from deer and wild boar meat, for example STEC O8:H9, O21:H21 and O27:H30, and O110:H31^48,49^. Phylogenetic analysis showed that only two STEC O27:H30 isolated were clonal. Highly similar STEC O27:H30 have been observed frequently in deer meat samples in Spain, suggesting an association between O27:H30 and deer^28^.

Taken together our data indicate that STEC present in game meat are genetically diverse, and that a subset of STEC may have the potential to cause extraintestinal infections in humans.

Finally, in this study, all the isolates carried chromosomal cephalosporinase genes and genes for RND efflux systems that are of clinical significance because they can confer resistance to third generation cephalosporins, aminoglycosides, and phosphonic acid derivatives, all of which are antimicrobials categorized by the World Health Organization (WHO) as critically important in human medicine^50^. These genes are ubiquitous in *E. coli*, however, over-expression of intrinsic AcrAB-TolC multi-drug efflux pump genes may lead to multidrug resistance and the likelihood of treatment failure in the case of a systemic infection with STEC/ExPEC^44,51^.

## Conclusions

This study identified game meat as a source of STEC, including STEC with serotypes, *stx* subtypes and other virulence traits that are associated with human disease.

Promoting awareness among hunters who handle game in the field, game meat manufacturers, and consumers is important to minimize the risk of exposure.

In addition, consumers and professionals within the food hospitality industry should be advised that products made from raw game meat such as tartare, carpaccio, and cured sausages are associated with a potential risk of infectious disease.

## Material and methods

### Sampling

An overview of the countries of origins and the suppliers of the game meat samples is given in Table 3. Samples originated from chamois (*Rupicapra rupicapra*), red deer (*Cervus elaphus*), roe deer (*Capreolus capreolus*), and wild boar (*Sus scrofa*), and were obtained during November 2021.

**Table 3:** Origin of 92 wild game meat samples from several European countries and from different suppliers.

Country of origin	Supplier			
	Butcher n = 21	Hunter n = 21	Processing plant^a^ n = 42	Swiss retail store n = 8
Austria n = 2				
Red deer	0	0	2	0
Croatia n = 1				
Red deer	0	0	1	0
Germany n = 8				
Red deer	0	0	0	3
Roe deer	0	0	0	3
Wild boar	0	0	0	2
Hungary n = 5				
Red deer	0	0	5	0
Poland n = 15				
Red deer	0	0	6	0
Roe deer	0	0	6	0
Wild boar	0	0	3	0
Slovenia n = 19				
Chamois	0	0	1	0
Red deer	0	0	6	0
Roe deer	0	0	7	0
Wild boar	0	0	5	0
Switzerland n = 42				
Chamois	0	1	0	0
Red deer	3	1	0	0
Roe deer	13	9	0	0
Wild boar	5	10	0	0

^a^The game meat processing plant is located in Slovenia.

The game meat processing establishment is located in Slovenia and processes domestic and imported hunted game animals and produces game meat cuts which are distributed in European countries (Table 3). During sample collection, lot numbers of the packed meat were noted to exclude that different samples originated from the same animal.

### Screening for *stx* genes

Each sample (10 g) was enriched at a 1:10 ratio in Enterobacteriaceae enrichment (EE) broth (Becton, Dickinson, Heidelberg, Germany) for 24 h at 37 °C. One loopful of each of the enrichment cultures was cultured on sheep blood agar (Difco™ Columbia Blood Agar Base EH; Becton Dickinson AG, Allschwil, Switzerland) using the streak-plate method. The resulting colonies were suspended in 2 ml 0.85% NaCl. Samples were then screened by real-time PCR for *stx1* and *stx2* using the Assurance GDS^®^ for Shiga Toxin Genes (Bio Control Systems, Bellevue, WA, USA).

### Recovery of STEC

In the event of a *stx* positive PCR result, one loopful of suspension was streaked onto STEC Chromagar plates (CHROMagar, Paris, FR) and Brolacin agar plates (Bio-Rad, Hercules CA, USA) to get single colonies. The plates were incubated at 37 °C overnight. From each plate, 20–180 individual colonies were picked (mauve colonies on STEC Chromagar plates; yellow colonies on Brolacin Agar plates) and suspended in 0.5 ml 0.85% NaCl. The suspensions were pooled in groups of material from ten colonies and screened for *stx1 and stx2* genes by real-time PCR (LightCycler R 2.0 Instrument, Roche Diagnostics Corporation, Indianapolis, IN, USA) using the QuantiFast Multiplex PCR Kit (Qiagen, Hombrechtikon, Switzerland) according to the guidelines of the European Union Reference Laboratory (EURL)^52^. In the event of a positive PCR result for *stx1* or *stx2*, the pool was taken apart and the ten colonies were tested again individually. From plates yielding more than one *stx1* and/or *stx2* positive colony, one presumptive STEC isolate was randomly chosen for subsequent characterisation by whole genome sequencing (WGS) analysis. If the screening results indicated colonies with different *stx* types, the different corresponding colonies were included in the further analysis.

### DNA extraction and whole genome sequencing

Isolates were grown on sheep blood agar at 37 °C overnight prior to DNA isolation using the DNA blood and tissue kit (Qiagen, Hombrechtikon, Switzerland). The DNA libraries were prepared using a Nextera DNA Flex Sample Preparation Kit (Illumina, San Diego, CA, USA). Whole genome sequencing was performed on an Illumina MiniSeq Sequencer (Illumina, San Diego, CA, USA). The Illumina-read files passed the standard quality checks using the software package FastQC 0.11.7 (Babraham Bioinformatics, Cambridge, UK) and were assembled using the Spades 3.14.1 based software Shovill 1. 1.0^53^, using default settings. The assembly was filtered, retaining contigs > 500 bp and annotated using the NCBI prokaryotic genome annotation pipeline^54^. Stx types were determined by an in silico PCR using the perl script "in_silico_pcr" (https://github.com/egonozer/in_silico_pcr) with the option "-m, allow one mismatch" activated and primer sets described in the EURL manual for *stx* genes detection^55^. The O- and H-types were identified using SerotypeFinder 2.0^56^. The sequence type (ST) of each strain was determined based on seven housekeeping genes using the tool "MLST"^57^using PubMLST as database (https://pubmlst.org/)^58^. The genetic relatedness of the isolates was assessed through core genome MLST (cgMLST) analyses using the Ridom SeqSphereC + software version 5.1.0 (https://www.ridom.de/seqsphere/). A minimum spanning tree (MST) was generated for visualization with the threshold for cluster identification set to ≤ 10 alleles between a pair of isolates, according to the Ridom SeqSphereC+ software. The virulence gene profiles and antimicrobial resistance genes were determined using VirulenceFinder 2.0^59^ and Resistance Gene Identifier (RGI) 4.2.2^60^. Subtilase cytotoxin A and B subunit genes and subtilase cytotoxin subtypes *subAB1* and *subAB2* were determined using Abricate^61^ with standard settings and an in-house made database containing nucleotide sequences of *subAB1* genes from *E. coli* 98NK2 (Acc. No. AY258503) and *subAB2* genes from *E. coli* ED32 (Acc. No. JQ994271). Presence of the adhesin gene *saa* was determined using tblastn with the Saa protein as input^62^ and the sequenced genomes as query. A cut off of > 70% identity with a 70% alignment rate was applied.

## Supplementary Information

Supplementary Table S1.

## Data Availability

This Whole Genome Shotgun project has been deposited at DDBJ/ENA/GenBank under the accessions JAPMME000000000 to JAPMNE000000000. The versions described in this paper are versions JAPMME000000000 to JAPMNE000000000. Accession numbers for the individual isolates from this study can be found as Supplementary Table S1 online. The BioProject number is PRJNA903888.
