# Supplementary material for: Whole genome sequence-based characterisation of Shiga toxin-producing Escherichia coli isolated from game meat originating from several European countries
Source: Sci Rep. 2023 Feb 24;13:3247. doi: 10.1038/s41598-023-30333-4 (PMC9957979; doi:10.1038/s41598-023-30333-4)
Supplement: Supplementary file 1 — Supplementary Table S1. [file 41598_2023_30333_MOESM1_ESM.docx]

**Supplementary Information**

**Whole genome sequence-based characterisation of Shiga toxin-producing *Escherichia coli* isolated from game meat originating from several European countries**

Magdalena Nüesch-Inderbinen ^a^ *, Andrea Treier ^a^, Marc J.A. Stevens ^a^, Roger Stephan ^a^

^a^ Institute for Food Safety and Hygiene, Vetsuisse Faculty, University of Zurich, Zurich, Switzerland

*Corresponding author: magdalena.nueesch-inderbinen@uzh.ch

**Table S1:** Summary of WGS data of 27 Shiga toxin-producing *Escherichia coli* isolated from game meat.

| **Strain ID** | **Sample ID** | **BioProject ID** | **Genome size (bp)** | **No. contigs** | **Accession No.** |
| --- | --- | --- | --- | --- | --- |
| B58 | W58 | PRJNA903888 | 4787193 | 93 | JAPMNA000000000 |
| B42 | W42 | PRJNA903888 | 5400325 | 189 | JAPMMT000000000 |
| C96-1 | W96 | PRJNA903888 | 5680166 | 295 | JAPMMK000000000 |
| C98-3 | W98 | PRJNA903888 | 5597429 | 231 | JAPMMG000000000 |
| B19-24 | W19 | PRJNA903888 | 5484883 | 240 | JAPMND000000000 |
| C15-2 | W15 | PRJNA903888 | 5592798 | 262 | JAPMMW000000000 |
| B20-22 | W20 | PRJNA903888 | 5592247 | 259 | JAPMNC000000000 |
| B86-6 | W86 | PRJNA903888 | 5537153 | 284 | JAPMMX000000000 |
| C81-2 | W81 | PRJNA903888 | 5378237 | 181 | JAPMMO000000000 |
| B37-47 | W37 | PRJNA903888 | 5587780 | 208 | JAPMMU000000000 |
| C96-6 | W96 | PRJNA903888 | 5471125 | 162 | JAPMMJ000000000 |
| C97-4 | W97 | PRJNA903888 | 5569190 | 261 | JAPMMI000000000 |
| C99-5 | W99 | PRJNA903888 | 5710562 | 188 | JAPMME000000000 |
| B62-1 | W62 | PRJNA903888 | 5725912 | 260 | JAPMMZ000000000 |
| B42-3 | W42 | PRJNA903888 | 5639361 | 257 | JAPMNB000000000 |
| C84-1 | W84 | PRJNA903888 | 5586555 | 273 | JAPMMN000000000 |
| C69-1 | W69 | PRJNA903888 | 5536330 | 178 | JAPMMR000000000 |
| C36-16 | W36 | PRJNA903888 | 5718731 | 256 | JAPMMV000000000 |
| C67-4 | W67 | PRJNA903888 | 5552230 | 239 | JAPMMS000000000 |
| C91-1 | W91 | PRJNA903888 | 5468217 | 231 | JAPMML000000000 |
| C99-3 | W99 | PRJNA903888 | 5638614 | 285 | JAPMMF000000000 |
| C79-1 | W79 | PRJNA903888 | 5631436 | 200 | JAPMMP000000000 |
| C89-1 | W89 | PRJNA903888 | 5525861 | 227 | JAPMMM000000000 |
| B75-8 | W75 | PRJNA903888 | 4995229 | 117 | JAPMMY000000000 |
| B16-28 | W16 | PRJNA903888 | 5181496 | 141 | JAPMNE000000000 |
| C73-1 | W73 | PRJNA903888 | 5722661 | 216 | JAPMMQ000000000 |
| C98-1 | W98 | PRJNA903888 | 5746767 | 331 | JAPMMH000000000 |
